# Whole-exome sequencing reveals GRHPR gene mutation in a 4-year-old girl with chronic kidney disease: a case report

**DOI:** 10.1097/MS9.0000000000004596

**Published:** 2025-12-18

**Authors:** Bakhtawar Farooq, Zahid H. Qureshi, Madeeha S. Lodhi, Muhammad Faisal, Esha Khan, Muddassir Khalid

**Affiliations:** a Institute of Molecular Biology and Biotechnology, The University of Lahore, Lahore, Pakistan; b Department of Biochemistry, Nishtar Medical University, Multan, Pakistan; c Department of Physiology, Multan Medical and Dental College, Multan, Pakistan; d Department of Biotechnology, MNS-University of Agriculture, Multan, Pakistan; e Department of Medicine, Nishtar Medical University, Multan, Pakistan

**Keywords:** case report, chronic kidney disease, CKD, genetics, WES, whole-exome sequencing

## Abstract

**Introduction and importance:**

Chronic kidney disease (CKD) is recognized as one of the leading causes of human death. This case highlights the importance of whole-exome sequencing (WES) and genetic diagnosis in families.

**Case Presentation:**

A 4-year-old girl (subject) was diagnosed with CKD along with her father, grandfather, and grandmother. The subject was first evaluated at 4 years of age because of black urine problem. On early diagnosis, it was kidney stones. She was suspected to be affected with kidney disorder by ultrasound and is being evaluated for the presence genetic variations by WES. We identified a missense variation in *GRHPR* gene c.494 G>A in subject, and her 27-year-old father was also identified with the same variation in the *GRHPR* gene. On follow-up, kidney stones were removed via extracorporeal shock wave lithotripsy (ESWL). Following ESWL sections, the subject was stable and sent home.

**Clinical discussion:**

Genetic diagnoses are essential for identifying diseases, which aid in early diagnosis and control to spread within families. This case highlights the value of advanced genetic testing in nephrology for precision diagnosis and family risk assessment.

**Conclusion:**

A multidisciplinary approach and timely intervention in CKD are vital to prevent complications in families.

## Introduction

Chronic kidney disease (CKD) is a global health concern marked by progressive renal function decline, primarily caused by diabetes, hypertension, heart issues, obesity, family history, and smoking^[1]^. CKD is characterized by a decrease in estimated glomerular filtration rate (eGFR) of less than 60 mL/min/1.73 m^2^ and kidney damage markers presented for more than 3 months^[2]^. The prevalence of CKD in families is rising, but awareness of CKD is low^[3]^. The progression of CKD is significantly impacted by an underlying genetic disorder^[3]^. The diagnosis of monogenic kidney disorders is essential, as it can profoundly influence a patient’s prognosis and clinical management, including the potential for targeted treatments^[4]^. Primary Hyperoxaluria Type 2 (PH2), caused by mutations in the *GRHPR* gene, disrupts the normal metabolism of glyoxylate – metabolism characterized by overproduction of oxalate – leading to oxalate nephropathy^[5]^. Whole-exome sequencing (WES) targets protein-coding regions for disease-associated variants and allowing comprehensive screening of kidney disease-related genes^[6]^. Early diagnosis halts progression of life-threatening disease^[5]^. A 4-year-old CKD girl with positive family history and with no genetic diagnosis was presented to our hospital, which later turned out to be a case of PH2 oxalate nephropathy with a mutation in *GRHPR* gene. The aim of this case report is to highlight the importance of genetic diagnosis and to explore a rare case of CKD patient with PH2. Therefore, clinicians can recognize the rare and subtle signs of PH2 and understand the complex interplay between the genetic mutation and the progressive kidney disease. The study has been reported in line with the SCARE 2025 checklist “[Kerwan A, Al-Jabir A, Mathew G, Sohrabi C, Rashid R, Franchi T, Nicola M, Agha M, Agha RA. Revised Surgical CAse REport (SCARE) guideline: An update for the age of Artificial Intelligence. Premier Journal of Science 2025:10;100 079.]”^[7]^.

‘This case report has been reported in line with the SCARE checklist [Kerwan A, Al-Jabir A, Mathew G, Sohrabi C, Rashid R, Franchi T, Nicola M, Agha M, Agha RA. Revised Surgical Case Report (SCARE) guideline: An update for the age of Artificial Intelligence. Premier Journal of Science 2025:10;100 079.]^[8]^.

## Case presentation

**Figure 1.**
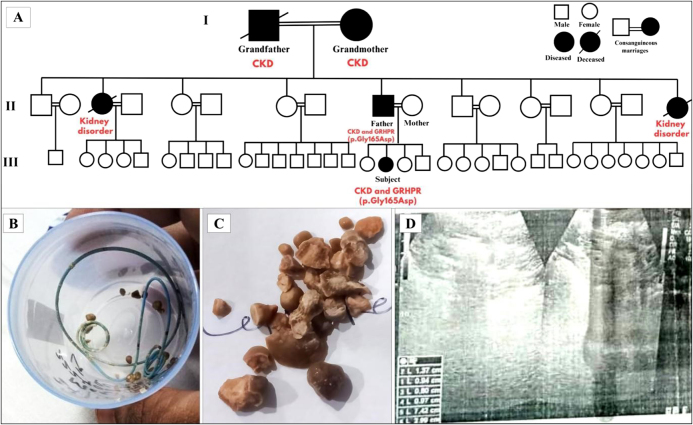
Comprehensive analysis of family history and medical details of the subject. These figures integrates genetic and clinical data, featuring the family pedigree (A), the size, shape, and color of the subject kidney stones (B and C), and the ultrasound report of subject (D).

The subject was a 4-year-old girl who was diagnosed with CKD on hospital admission. She was the second of four children born to consanguineous parents. Her father, grandfather, and grandmother had CKD while no other family members, including the mother and siblings, were diagnosed with CKD. Other than these two father’s sisters (paternal aunt) were diagnosed and died with severe kidney disorder. Grandparents and all father siblings were married by consanguineous marriages. The pedigree of subject family is presented in Fig. 1A. The father had kidney issues since his childhood. The subject was first evaluated at 4 years of age because of black urine problem. On early diagnosis, it was kidney stone and renal disorders presented by ultrasound results. The high-resolution abdominal ultrasound revealed that the left DJ stent was in-place, along with multiple renal calculi of variable sizes, non-obstructive right renal lithiasis, and features of recurrent cystitis (Fig. 1).

The details of laboratory diagnosis of subject are mentioned in Table 1. The elevated serum creatinine (1.2 mg/dL) and low eGFR (73 mL/min/1.73 m^2^) confirm the presence of stage 2 CKD. Her father was also diagnosed with stage 2. Subject exhibits anemia with low hemoglobin (9.7 g/dL), hematocrit (34%), mean corpuscular volume (MCV; 69.2 fL), mean corpuscular hemoglobin (MCH; 19.8 pg), and mean corpuscular hemoglobin concentration (MCHC; 28.6 g/dL). The white blood cells count is significantly elevated, and the relatively low percentage of lymphocytes suggests a strong inflammatory process. Serology assays showed a positive hepatitis C virus. The low serum bicarbonate (18.8 mmol/L) indicates metabolic acidosis, a common complication of CKD. Subject was suspected to be affected with kidney stones and the details of stone are mentioned in Table 2. The chemical composition of renal stone shows the presence of 90% calcium oxalate monohydrate and 10% of struvite.

**HIGHLIGHTS**

- Whole-exome sequencing identified a pathogenic GRHPR mutation causing Primary Hyperoxaluria Type 2 in a 4-year-old with chronic kidney disease (CKD).
- The same mutation in the father confirmed hereditary kidney disease.
- Clinical findings showed stones, anemia, acidosis, and stage 2 CKD.
- In silico and American College of Medical Genetics and Genomics analyses supported the variant’s pathogenicity.
- Early genetic diagnosis enabled targeted management and family screening.

**Table 1:** Laboratory diagnosis

Test	Result	Unit	Normal range
White blood cells	26.42↑	10^3^/µL	5–12
Lymphocytes	19.4↓	%	20–40
Red blood cells	4.91	10^6^/µL	4–5.20
Platelet	4.93↑	10^3^/µL	100–300
Hemoglobin	9.7↓	g/dL	12–15.5
Hematocrit	34↓	%	35–49
Plasma PTH (intact)	20	pg/mL	16–87
Mean corpuscular volume	69.2↓	fL	82–95
Mean corpuscular hemoglobin	19.8↓	pg	27–31
Mean corpuscular hemoglobin concentration	28.6↓	g/dL	32–36
Urinary oxalate	20	mg/24 hr	13–34
Urine uric acid	80.50↓	mg/24 hr	200–1000
Urine protein	110	mg/24 hr	0–150
Urine calcium	12.20↓	mg/24 hr	100–300
Serum sodium	138	mmol/L	136–145
Serum potassium	4.7	mmol/L	3.5–5.1
Serum chloride	102	mmol/L	98–107
Serum bicarbonate	18.8↓	mmol/L	22–28
Serum calcium	9.6	mg/dL	8.6–10.2
Serum uric acid	1.2↓	mg/dL	2–5
Bood urea	36	mg/dL	20–45
Serum creatinine	1.2↑	mg/dL	0.4–1.1
Serum eGFR	73↓	mL/min/1.73 m^2^	90 or higher
Hepatitis B virus	Negative	—	—
Hepatitis C virus	Positive	—	—

**Foot Note:** ↑: Increased level, ↓: Decreased level.

**Table 2:** Physical characteristic and chemical composition of renal stone

Physical characteristic and chemical composition	Result
Source of stone	Renal stone
Number	>5 stones
Size	2.640 gm
Weight	0.2–0.5 cm
Shape	Irregular
Color	Light brown
Calcium oxalate monohydrate	90%
Struvite	10%

### Investigations and genetic testing

Furthermore, subject was being evaluated for the presence of any pathogenic/benign variations. WES was conducted at lab. Identified variants were confirmed via Sanger sequencing and classified according to the international guidelines of the American College of Medical Genetics and Genomics (ACMG) via GeneBe (https://genebe.net/). A homozygous missense variation in exon 6 of the *GRHPR* gene (chr9:37 429 729 G>A) was identified in a 4-year-old female patient that substitutes glycine for aspartate at codon 165 (p.Gly165Asp) lead to hyperoxaluria primary type II (OMIM#260 000). The variant was classified as likely pathogenic per ACMG criteria. The subject father was also identified with the same variation in the *GRHPR* gene. The results of additional finding found homozygous missense variation in *GNE* gene (chr9:g.36217445C>T) in the subject’s father, causing a methionine-to-valine substitution (p.Val696Met), which has been associated with Nonaka myopathy (OMIM#605 820). Based on ACMG criteria, this variant was classified as variant of uncertain significance.

### KEGG pathways

The pathways of *GRHPR* gene in human were studied using website of KEGG pathways (https://www.genome.jp/kegg/pathway.html). The pyruvate, glycine, serine, and threonine, as well as glyoxylate and dicarboxylate metabolism, illustrate the crucial role of the *GRHPR* gene. Theses pathways shows that GRHPR reduces glyoxylate to glycolate and hydroxypyruvate to D-glycerate using NADPH, preventing excessive glyoxylate accumulation and minimizing oxalate formation.

### In silico assessment of genetic variation

**Figure 2.**
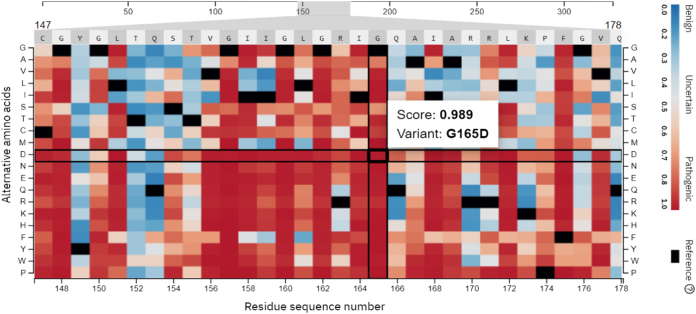
The AlphaMissense heatmap of predicted pathological effects of mutation saturation in Glyoxylate Reductase/Hydroxypyruvate Reductase (*GRHPR*) gene.

*In silico* prediction tools like Mutation Taster, SIFT, PolyPhen2, FATHMM, and I-Mutant2 were used to assess the effect of genetic variation on gene structure and function. Genetic variation in the *GRHPR* gene was flagged by SIFT and classified as probably damaging by PolyPhen2 and FATHMM. I-Mutant2 indicated a decrease in protein stability and indicating a pathogenic or likely pathogenic variant with a score of 0.98 by AlphaMissense heat map (Fig. 2).

### Protein-protein interaction network analysis

**Figure 3.**
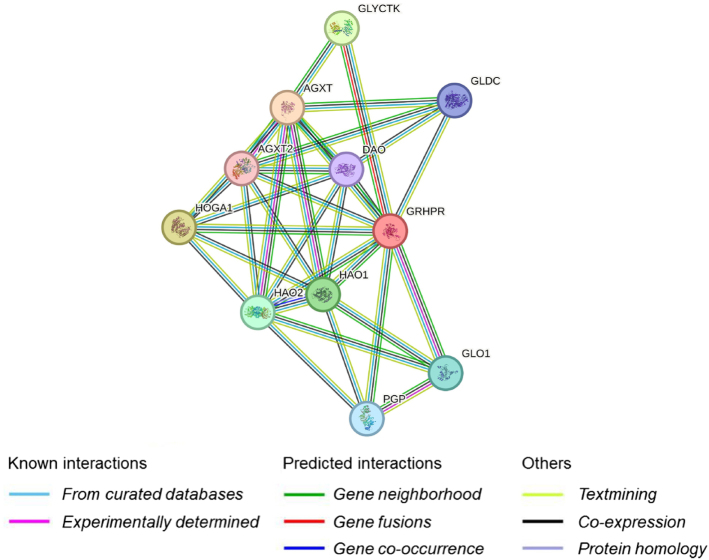
Protein-protein interaction analysis of GRHPR protein by STRING.

Protein-protein interaction (PPI) network analysis was performed using the online gene/protein interaction retrieval website STRING (http://www.string-db.org/). PPI network analysis revealed that the GRHPR protein directly interacts with AGXT, HOGA1, GLYCTK, HAO1, HAO2, GLO1, PGP, GLDO, DAO, and AGXT2, indicating potential functional relationships among 11 proteins, while edges indicated predicted functional and physical associations for each protein (Fig. 3).

### Outcomes and follow-up

After 6 months, subject presented with localized, moderate left flank pain. On follow up, four Extracorporeal Shock Wave Lithotripsy (ESWL) procedures were performed (shocks 2500, frequency 1.0) to remove numerous stones. ESWL is a non-invasive procedure that uses extracorporeal sound waves to break kidney stones into smaller pieces for natural passage in urine. Following these procedures, some residual stones remained. Following ESWL sections, the patient is stable and discharged to their home. This indicates a successful immediate post-procedure recovery.

## Discussion

This case highlights the critical role of WES in resolving genetic diagnostic in CKD. Here, we presented a 4-year-old CKD girl with homozygous missense variation in *GRHPR* gene that causes PH2. Similar *GRHPR* gene mutation was found in the father, indicating familial spread. These findings have profound implications for initiating crucial familial screening. Previous researcher reported that PH2 is a genetic disorders due to mutation in *GRHPR* gene^[9,10].^ Chatterjee *et al* identified mutation of *GRHPR* gene (c.494 G>A) which is consistent with our study and reported that mutation in *GRHPR* gene leads to the reduction of glyoxylate to glycolate and its subsequent reconversion back to glyoxylate, which is the dual enzyme activity of glyoxylate reductase and hydroxy pyruvate reductase which leads to kidney stones and HP2^[11].^

Current study lab results reported low level of hemoglobin, hematocrit, MCV, MCH, and MCHC in subject that indicate anemia. Anemia is a frequent complication of CKD^[12].^ The chemical composition of the renal stone in subject revealed calcium oxalate monohydrate and struvite stones. Calcium oxalate is a predominant component of renal stones and exists as monohydrate and dihydrate forms^[13]^. Risk factors for oxalate crystal formation include low urine volume, high calcium and oxalate excretion, dietary influences, vitamin D excess, intestinal bypass surgery, and metabolic disorders^[13,14]^.

The *GRHPR* gene is crucial in preventing excess oxalate formation^[15]^, which is also illustrated by KEGG pathways mentioned in our study. Mutations lead to reduced function, causing glyoxylate accumulation, which converts to oxalate^[16]^. This poorly soluble substance binds with calcium, forming insoluble calcium oxalate crystals that deposit in renal tissue, linking *GRHPR* variations to kidney oxalate accumulation^[15,16]^. Low serum bicarbonate (reported in this report) is a common complication in CKD, resulting from impaired acid excretion. As CKD progresses, metabolic acidosis occurs, decreasing blood pH and serum bicarbonate levels, leading to potential health issues^[17]^. Untreated acidosis worsens kidney function, increases muscle wasting, causes bone demineralization, and promotes inflammation. Metabolic acidosis may even cause death of patients^[18]^. Furthermore, the results of PPI show that GRHPR directly interacts with AGXT, HOGA1, GLYCTK, HAO1, HAO2, GLO1, PGP, GLDO, DAO, and AGXT2 proteins. Corredor *et al* reported that GLO1 is associated with CKD progression^[8]^. As GRHPR has been shown to be associated with CKD-associated protein by PPI; therefore, it shows that GRHPR is associated with CKD. Therefore, the case underscores WES’s role in pediatric nephrology, facilitating conclusive diagnoses, timely treatment decisions, and proactive management of hereditary kidney diseases.

## Conclusion

This case study highlights the WES in pediatric nephrology, especially the diagnosis of uncommon genetic conditions. WES reveals a pathogenic *GRHPR* gene mutation in CKD patient that leads to PH2. In addition to improving the child’s prognosis, this strategy makes it easier for family members to have preventive screenings and counseling, which eventually opens the door for early management of hereditary kidney illnesses.

TITAN Guidelines: This manuscript complies with TITAN Guidelines, 2025, declaring no use of AI^[19]^.

## Data Availability

Variant submit to NCBI ClinVar accession numbers SCV006297958 (*GRHPR* gene) and SCV006297959 (*GNE* gene).

## Peer and provenance statement

Not commissioned, externally peer-reviewed.
